# Formation and Structural Characteristics of Heating-Induced Amyloid Fibrils Derived from Rice Albumin at Different pH Values

**DOI:** 10.3390/foods14173069

**Published:** 2025-08-30

**Authors:** Ting Li, Li Wang

**Affiliations:** 1 School of Food Science and Technology, Jiangnan University, Lihu Road 1800, Wuxi 214122, China; 2 National Engineering Research Center for Cereal Fermentation and Food Biomanufacturing, Jiangnan University, Lihu Road 1800, Wuxi 214122, China

**Keywords:** rice albumin, heat treatment, amyloid fibrils, assembly behavior, structural characterization

## Abstract

The comparison of rice albumin (RA) after heat treatment at neutral and acidic conditions was investigated in this study. Compared to the decreased thioflavin T (ThT) intensity of RA at pH 2 during heating, the ThT intensity of RA at pH 7 increased throughout the process of fibrillization. After fibrillization, the ThT intensity of RA at pH 7 was significantly increased by 27%, 38% and 35% at the protein concentrations of 1%, 2% and 4%, respectively. In addition, worm-like fibrils with a contour length of 100–300 nm were formed after heating at neutral conditions, accompanied by an increased average particle size and structural re-arrangement. Furthermore, the fibril formation at pH 7 involved the enhancement of an ordered β-sheet structure. However, only spherical agglomerate with a larger average particle size (>2000 nm) was observed when RA was heated at pH 2, because excessive hydrolysis destroyed the fibril-core sequences of RA. Additionally, the low solubility and high hydrophobicity of RA at pH 2 were not conducive to the formation of fibrils. In a word, a neutral environment is suitable for RA-based fibril formation, which provides a new insight for its future uses in food products.

## 1. Introduction

Amyloid fibrils have been traditionally described as pathological aggregates associated with human neurodegenerative diseases. However, it is increasingly becoming clear that most proteins contain a core sequence that could assemble into amyloid fibrils, including food proteins [1]. Xu et al. proved that food protein-based amyloid fibrils can be regarded as safe ingredients through in vitro and in vivo studies [2]. In addition, food protein fibrils have attracted significant attention due to their desirable functional properties and multi-applications in the food industry [3]. Up to now, the fibrillization mechanism and application regarding animal proteins, such as lysozyme and β-lactoglobulin, have been well researched. However, the growth of the global population and increased living standards lead to a continuous increment in the need for protein. Moreover, livestock farming resulted in many environmental problems. Due to sustainability concerns, the transition to plant-based protein for producing amyloid fibrils has become increasingly critical [4]. Therefore, elucidating the formation and structural characteristics of plant protein-based amyloid fibrils is important.

Rice is an important staple food and a primary source of protein in the world. Broken rice is the main byproduct during the rice milling process, and 36.6% of broken rice globally in 2020 was used for non-food purposes, resulting in the inefficiency of resource utilization [5]. Consequently, the comprehensive utilization of broken rice is of great significance. Broken rice contains abundant nutrients, such as starch and protein. In recent decades, rice protein has obtained extensive attention due to its superior quality, such as hypoallergenic properties, balanced amino acid composition, high protein digestibility and biological value [6]. Therefore, the development of rice protein from the byproduct of rice processing (broken rice) is conducive to improving the feasibility of resource utilization and providing economic benefits. However, the application of rice protein is limited due to its high hydrophobicity and low solubility at neutral pH. Our previous studies proved that the amyloid fibrils of rice protein were manufactured after heating at pH 2 and 90 °C, and the functional properties of proteins were improved due to specific structural features [7]. Considering that most food applications were based on neutral conditions, previous work regarding rice amyloid fibrils formation only focused on extremely acidic conditions, which were not suitable for most food products. Huyst et al. found that ovalbumin can form amyloid fibrils at neutral conditions, and ovalbumin fibrils can be used in emulsion systems [8], proving a promising insight for food applications. In addition, the amyloid fibrils from bovine serum albumin and β-lactoglobulin can be formed at neutral pH [9]. As reported, rice bran protein fibrils exhibited differences in structures and properties at pH 2 and pH 7 [10]. Rice albumin (RA) is a major water-soluble protein fraction in rice protein at neutral solution, containing abundant glutamic acid (Glu) and aspartic acid (Asp), which makes RA a superior building block for producing fibrils in a neutral environment. Moreover, RA-based fibrils (RAFs) would be a promising ingredient for food applications. To the best of our knowledge, the albumins derived from animal proteins, such as ovalbumin, α-lactalbumin and bovine serum albumin, exhibited differences in structures and properties at different pH values [1,11,12]. However, there were no studies focused on plant-based albumin fibrillization after pH adjustment. Therefore, the comparison of rice albumin fibrils at different pH values will help to enhance the understanding of the effects of neutral and acidic condition-mediated protein fibrillization, and high-value utilization of RA in the food field.

This study aimed to elucidate the formation and structural characteristics of heating-induced amyloid fibrils derived from RA. The comparison of RA heated at neutral and acidic conditions was also investigated. Overall, this study may contribute to the exploitation and application of RAFs as a novel ingredient for food-related products.

## 2. Materials and Methods

### 2.1. Materials

The rice was purchased from Jinwanxian Co., Ltd. (Anqing, China). The Mini-PROTEAN TGX Gel, Laemmli buffer and maker with molecular weight (Mw) of 10–250 kDa were procured from Bio-Rad Laboratories Inc. (Hercules, CA, USA). The Coomassie Blue Staining Solution and bicinchoninic acid (BCA) assay kit were acquired from Beyotime Institute of Biotechnology Co., Ltd. (Shanghai, China). Thioflavin T (ThT) dye was obtained from TCI (Tokyo, Japan). The 8-anilino-1-naphthalenesulfonic acid (ANS) (97%) was purchased from Sigma-Aldrich (St. Louis, MO, USA). Other reagents were all analytical unless otherwise stated, which were obtained from Sinopharm Chemical Co., Ltd. (Shanghai, China).

### 2.2. Extraction of Rice Albumin (RA)

As reported, rice proteins contain albumin (water-soluble), globulin (salt-soluble), prolamin (alcohol-soluble) and glutelin (alkali/acid-soluble) fractions based on solubility classification. According to a previous study, the alkaline solution of rice protein was adjusted to neutral conditions (pH 7) to separate water-soluble albumin and other insoluble fractions [13]. Initially, the sieved rice flour (80 mesh) was mixed with distilled water at a ratio of 1:10, and the pH was adjusted to pH 12. The resulting suspension was stirred for 2 h and then centrifugated at 5000× *g* for 15 min. Afterwards, the solution was adjusted to pH 7 and then centrifugated at 5000× *g* for 15 min to collect the albumin fraction (supernatant). After that, the obtained homogenous solution was precipitated after adjusting to pH 4.1 (isoelectric point). Finally, the albumin fraction was adjusted to pH 7 and dialyzed for 24 h in distilled water using a semipermeable membrane (Spectra/Por dialysis membrane, MWCO of 3.5 kDa) and freeze-dried for 3 days to collect RA powder.

### 2.3. Formation of Rice Albumin-Based Amyloid Fibrils (RAFs) at Different pH Values

The RAFs were prepared according to our previously published study with some modifications [7]. Firstly, the RA powder was dispersed in distilled water and hydrated for 2 h to obtain samples with various protein concentrations, i.e., 1%, 2% and 4% (*w*/*v*). The protein concentration of suspensions was determined by the Kjeldahl method [14]. Then, the sample solutions were adjusted to pH 2 and pH 7. In general, with the increase in ionic strength, the ordered extension of fibrils was accelerated, but the extension of fibrils was inhibited beyond a certain ionic strength. After consulting the literature, the ionic strength of 150 mM was widely used in previous studies [15,16,17]. In addition, according to our previous studies, the suitable ionic strength (150 mM) accelerated the fibrils assembly. Therefore, the NaCl stock solution was added to RA dispersions to achieve an ionic strength of 150 mM. After that, these sample solutions were hydrated at 4 °C overnight. The resulting samples were injected into bottles with sealed gaskets to avoid evaporation during heating. Subsequently, the samples were heated at 90 °C for 24 h with constant stirring of 300 rpm and then taken out during fibrillization (0, 0.5, 1, 2, 4, 6, 8, 10, 12, 24 h) [18,19]. Subsequently, these samples were cooled in an ice bath. Finally, these obtained samples were stored in a refrigerator (4 °C) for further determination.

### 2.4. ThT Fluorescence Intensity

According to the method of Li et al., the ThT assay was used to observe the formation of amyloid fibrils with some modifications [20]. To prepare the ThT stock solution, ThT powder was dissolved in 10 mL phosphate buffer (10 mM, pH 7) and filtered through a 0.45 μm filter. And the ThT stock solution was diluted 50 times to generate the ThT working solution. After that, the obtained RAFs samples (50 μL) during heating (0–24 h) were added to the ThT working solution (4 mL) and then reacted for 2 min in the dark. Next, the ThT intensities of the RA and RAFs were measured using a fluorescence spectrophotometer (F-7000, Hitachi Co., Tokyo, Japan) at an excitation of 440 nm and emission of 490 nm with excitation and emission slit widths of 5 nm. The corrected ThT intensity was collected after subtracting the fluorescence intensity of the ThT working solution.

### 2.5. Atomic Force Microscopy (AFM)

RAFs solutions at different pH values were diluted to 0.02 mg/mL using distilled water at the corresponding pH before depositing on freshly cleaved mica. Then, the diluted samples (10 μL) were deposited on the surface of mica. After drying at 25 °C, the images of the samples were taken using a Dimension Icon atomic force microscope (Bruker Corp., Billerica, MA, USA) using the Scan Asyst mode and a resolution of 256 pixels per line. Finally, the collected images were analyzed using NanoScope Analysis version 1.90 (Bruker Corp., Billerica, MA, USA).

### 2.6. Sodium Dodecyl Sulfate Polyacrylamide Gel Electrophoresis (SDS-PAGE)

The SDS-PAGE experiment was conducted according to the method in a previous study [21]. Firstly, the heated samples were mixed with a loading buffer that contained the Laemmli sample buffer and DL-dithiothreitol. The prepared samples (2 mg/mL) were heated for 5 min at 100 °C. The precast polyacrylamide gels were immobilized in an electrophoresis tank, and then the electrophoresis buffer was poured into the tank. After that, the prepared samples and maker (10 μL) were added to precast gels, and the electrophoresis was performed at 200 V for 40 min. After that, the gels were stained using Coomassie Blue Fast Staining Solution and destained using distilled water. The images of the gels were collected using a gel imaging system (Tanoon 4100, Shanghai, China).

### 2.7. Far-UV Circular Dichroism (CD) Spectra

The samples were diluted to 0.2 mg/mL using distilled water (pH 2) and then injected into a quartz cuvette with an optical path of 1 mm. The CD spectra ranging from 190 to 260 nm were carried out using a Chirascan V100 spectrometer (Applied Photophysics Ltd., Leatherhead, UK). The scanning speed and bandwidth were 100 nm/min and 1 nm, respectively. The samples were scanned in triplicate, and the CD spectrum of distilled water was recorded as background. The final CD spectra that had the subtracted background were analyzed using the BeStSel prediction algorithm [22].

### 2.8. Average Particle Size

RAF solutions were diluted to 0.2 mg/mL and transformed to an electrophoresis cell (DTS 1070) before measurement. The average particle size was tested using a Zetasizer Nano ZS (Malvern Instrument Ltd., Worcestershire, UK). The parameters of this test were shown as follows: scattering angle of 173°, room temperature of 25 °C and equilibration time of 120 s. Each experiment was repeated three times.

### 2.9. Solubility

The solubility of RA at different pH values was determined using the BCA method [23]. RA was diluted using 10 mM phosphate buffer with different pH values (pH 2 and pH 7) to a concentration of 1 mg/mL and then centrifugated at 5000× *g* for 10 min. The supernatant (20 μL) was mixed well with the working solution (200 μL) and maintained for 25 min at 37 °C. The resulting mixture was detected using a microplate reader at 562 nm (SH-1000, Hitachi, Tokyo, Japan). The absorbance of bovine serum albumin with a concentration of 0–1 mg/mL at 562 nm was recorded and plotted as the standard curve. The protein concentration of the samples was calculated by referring to the standard curve of bovine serum albumin. The solubility of samples was calculated through Equation (1).

(1) $$Solubility\;\left(\%\right)=\frac{C_{1}\times V_{1}}{m_{2}}\times 100\%\;$$

where C_1_ and V_1_ are the protein concentration and volume of supernatant, respectively, and m_2_ is the protein weight of the total sample.

### 2.10. Surface Hydrophobicity (H_0_)

The surface hydrophobicity of the samples was determined according to our previous studies [23]. The ANS solution (8 mM in 10 mM phosphate buffer, pH 7) was freshly made before measurement. The diluted samples (2 mL) with concentrations of 0.1, 0.2, 0.3, 0.4 and 0.5 mg/mL were mixed well with ANS (50 μL) and reacted for 2 min in the dark. Then, the mixed samples were measured at the excitation and emission of 390 and 470 nm. The calculated slope of the linear curve between the fluorescence intensity and concentration was H_0_.

### 2.11. Statistical Analysis

All assays were repeated three times, and the data were expressed as means ± standard deviations. The Statistical Product and Service Solutions (SPSS) Statistics 20 software was used to process and analyze the results. The differences between the samples were subjected to one-way analyses of variance (ANOVA) and Duncan test (*p* < 0.05).

## 3. Results and Discussion

### 3.1. ThT Fluorescence Intensity

**Figure 1:**
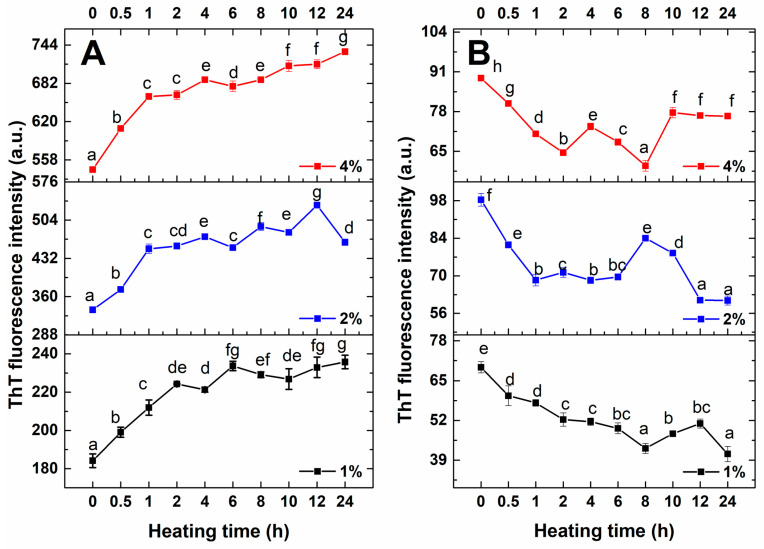
The ThT fluorescence intensity of RA after heating at different conditions ((**A**). pH 7, (**B**). pH 2). Data are exhibited as mean ± standard error. The different letters (a–g) mean a significant difference in the data (*p* < 0.05).

Amyloid fibrils are characterized by a cross-β sheet and steric zipper structures at the molecular level. ThT fluorescence is a gold standard method for detecting the specific cross-β sheet structure of amyloid fibrils, which is widely used to determine the kinetic growth during fibrillization [1]. The kinetic growth of amyloid fibrils from RA at different pH values (2 and 7) and protein concentrations (1%, 2%, 4%) is shown in Figure 1. As depicted in Figure 1A, the ThT intensity of RA at pH 7 varied as a function of heating time and enhanced throughout the process of fibrillization. The ThT intensity of RA at a concentration of 1% increased rapidly at the initial heating time (0–2 h), while the rapid growth phase of RA at 2% and 4% was shortened to 1 h. This result indicated that the growth rate of RAFs was improved with increased protein concentration. From 2 to 24 h, the ThT intensity of RA at a concentration of 1% increased slowly and reached the maximum value, illustrating that the fibril formation eventually reached the plateau stage. With increased heating time, the ThT intensity of RA at a concentration of 2% gradually increased and reached a maximum value at a heating time of 12 h. Similarly, the ThT intensity of RA at a concentration of 4% gradually increased with increased heating time and achieved the maximal intensity at a heating time of 24 h. After fibrillization, the ThT intensity of RA at pH 7 was increased by 27%, 38% and 35% at the protein concentrations of 1%, 2% and 4%, respectively. Compared to other concentrations, the fibrils achieved the highest fibrils yield with the shortest heating time at a protein concentration of 2%. Furthermore, the normalized ThT intensity was fitted, and the growth rate was calculated, which is displayed in Table S2 and Figure S1. It can be seen that the growth rate of RA was enhanced from 0.93 h^−1^ to 1.09–1.37 h^−1^ with increased protein concentration. The faster growth rate was achieved at a protein concentration of 2% (1.37 h^−1^), which was comparable to rice glutelin with different types of ions during fibrillization [24]. The maximum ThT intensity of RA at 2% was higher than that of peanut protein fibrils (~250 a.u.) [25], but was lower than that of pea globulin amyloid-like fibrils (~700 a.u.) [26] and soy protein fibrils (~1000 a.u.) [27]. Therefore, the optimized protein concentration at 2% was selected for further structural analysis.

Pearce et al. also observed that when heated at neutral pH, ovalbumin showed a rapid increase in ThT intensity, indicating the formation of amyloid fibrils [28]. The ThT intensity of RA at pH 2 is lower than at pH 7, indicating a lower level of fibril formation at the acidic condition. In addition, the ThT fluorescence signal of RA at pH 2 decreased with prolonged heating time, implying that pH 2 was not suitable for the assembly of RAFs. As previously reported, a protein with lower solubility was not prone to fibril formation [29]. Specifically, millet protein with solubility of 58% assembled into aggregates after heating at pH 2 [30]. Therefore, we hypothesized that the RA at pH 2 with lower solubility (32.53%, Table S1) was prone to form irregular aggregates, which was not beneficial for the assembly of amyloid fibrils [31]. Liu et al. also found that the formation of pea protein fibrils was influenced by different pH values [21].

### 3.2. Morphology Studies

**Figure 2:**
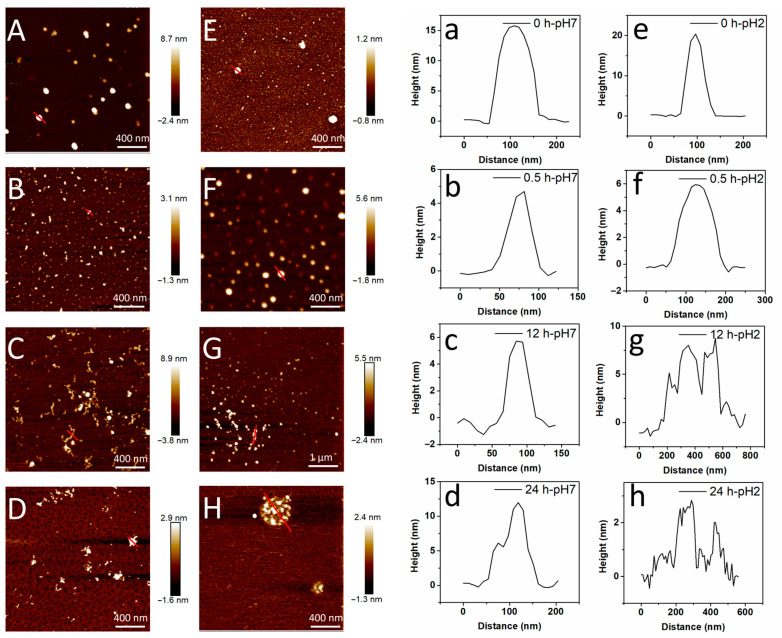
Morphology analysis of RA (2%) during fibrillization (**A**–**D**). AFM images of RA after heating for 0, 0.5, 12, 24 h at pH 7; (**E**–**H**). AFM images of RA after heating for 0, 0.5, 12, 24 h at pH 2; (**a**–**d**). Height distribution of RA after heating at pH 7 with different times (0, 0.5, 12, 24 h); (**e**–**h**). Height distribution of RA after heating at pH 2 with different times (0, 0.5, 12, 24 h). The red lines in AFM images (**A**–**H**) represent the cross sectional analysis of the samples.

AFM is widely used to analyze the morphology of amyloid fibrils at the nanoscale. The AFM images of RAFs induced by different pH values are shown in Figure 2. For unheated RA samples (0 h), fibrils were not observed (Figure 2A,E). In addition, the unheated RA at pH 7 exhibited spherical particles with a height of 15 nm (Figure 2A,a). In Table S3, after heating for 0.5 h, some short protofibrils with an average length of 78.83 nm began to be observed (Figure 2B), and the height of the sample was reduced to approximately 4.63 nm (Figure 2b), illustrating the partial denaturation and hydrolysis of protein. After heating for 6 h, some shorter flexible fibrils with an average contour length of 112.17 nm and an average height of 4.68 nm were displayed in Figure S2. With the extension of heating time (12 h), worm-like fibrils with the average contour length of 199.74 nm and height of 5.09 nm were formed (Figure 2C,c, Table S3), confirming that the protofibrils were elongated to flexible fibrils during the growth stage (0.5–12 h). In addition, the maximum contour length of 421 nm was displayed in RA after heating for 12 h, which was greater than lupin (~390 nm), soybean (~220 nm) and potato protein fibrils (~340 nm), but lower than fava bean (~720 nm), mung bean (~550 nm), oat (~760 nm) and rapeseed protein fibrils (~910 nm). And the average height of RA at pH 7 was raised from 4.63 to 5.09 nm when the heating time increased from 0.5 h to 12 h, which was similar to other plant amyloid fibrils (2.7–5.3 nm) [32]. However, the fibrils were aggregated into larger and irregular clusters with a height of approximately 12 nm after heating for 24 h, which was in line with the reduced ThT intensity at 24 h (Figure 2D,d). This illustrated that the fibril self-assembly was highly dependent on the extent of heating time. As reported, the protein fibrillization involved protein unfolding, elongation and maturation stages, in which the protein was hydrolyzed to smaller peptides and then self-assembled into amyloid fibrils [4]. Consistently, Zhang et al. also found that the protein particles of rice bran albumin gradually evolved into smaller rod-like fibrils and then formed longer linear fibrils with increasing heating time [33].

As shown in Figure 2E,e, spherical particles with a height of ~20 nm occurred in RA at pH 2. As the heating time prolonged (0.5 h), the RA particles were depolymerized into smaller particles with a height of ~6 nm (Figure 2F,f). As the heating process continued, multiple particles assembled into irregular aggregates (Figure S3) and beaded-like aggregates (Figure 2G,g), and eventually formed the spherical agglomerate (Figure 2H). Moreover, the particle size was reduced to approximately 3 nm after heating for 24 h (Figure 2h). In addition, Xu et al. observed the peanut protein aggregation after hydration treatment at pH 2, which inhibited the fibril-forming capacity and self-assembly process [25]. Overall, it was concluded that the structure of protein and fibrils was susceptible to pH changes [34]. And RA assembled into diverse morphologies at different pH values during heating, among which RA assembled into worm-like flexible fibrils at pH 7, but a spherical agglomerate was formed at pH 2. This phenomenon indicated that a suitable pH was necessary for fibril formation.

### 3.3. SDS-PAGE

**Figure 3:**
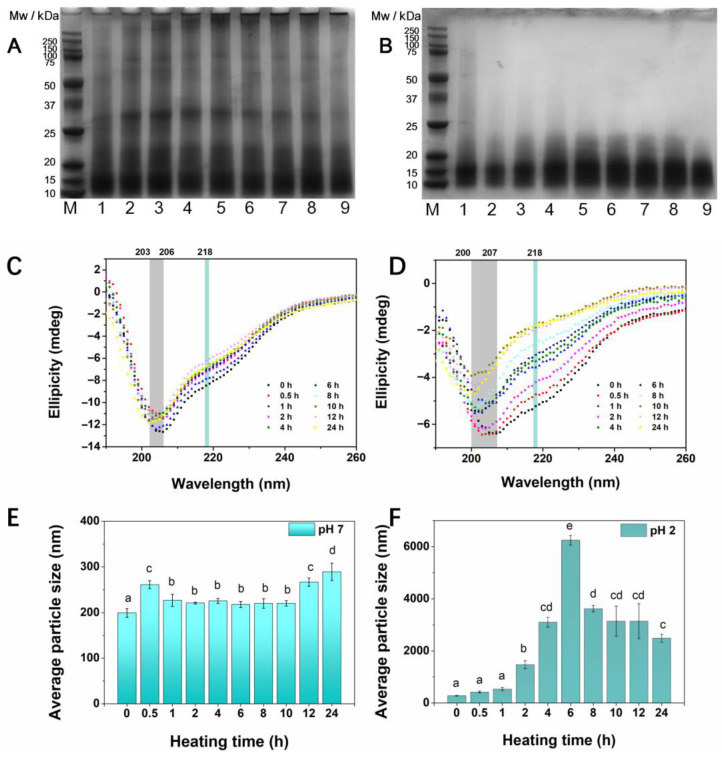
Structural characteristics of RA (2%) after heating at different pH values. (**A**,**B**) The SDS-PAGE of RA (2%) after heating at pH 7 and pH 2, respectively (M. maker, 1–9 samples heated for 0, 0.5, 1, 2, 4, 6, 10, 12, 24 h); (**C**,**D**) CD spectra of RA (2%) after heating at different pH values ((**C**). pH 7, (**D**). pH 2); (**E**,**F**) The average particle size of RA (2%) at different pH values during fibrillization ((**E**). pH 7, (**F**). pH 2). Data are exhibited as mean ± standard error. The different letters (a–e) mean a significant difference in the data (*p* < 0.05).

Previous reports proposed the monomer model and polypeptides model for the protein formation process [4]. For the monomer model, the protein monomers are misfolded during denaturation and activation and eventually form fibrillar aggregates. According to the polypeptides model, the spherical proteins are hydrolyzed to produce peptides and expose the amyloid cores, and then self-assemble into fibrillar aggregates during fibrillization [3]. Interestingly, the protein hydrolysis and self-assembly may occur simultaneously during fibrillization [25]. To investigate the protein hydrolysis process, the Mw distribution was detected by SDS-PAGE. The SDS-PAGE images of RA at different pH values are shown in Figure 3. As displayed in Figure 3A, RA at pH 7 exhibited a wide range of subunit compositions from 10 to 200 kDa and a major band at Mw of ~15 kDa, which was in line with previous reports [35]. In addition, the subunits at ~20 kDa and ~25 kDa were attributed to the contamination of rice globulin during protein extraction. Because minerals (NaCl) are present in the extraction solution after adjusting the alkaline solution to neutral conditions, the solubility of the globulin increased [36]. When the heating time ranged from 0 to 1 h, no distinct changes in subunit compositions were observed, indicating that the primary structure of RA remained stable during the initial phase of fibrillization. Other researchers also found that hemp seed protein retained their structural integrity at pH 7 after heating at 90 °C, from SDS-PAGE results [37]. With prolonged heating times (1–24 h), the bands with higher Mw (20–200 kDa) of RA at pH 7 faded away and shifted downward, finally reaching the Mw < 20 kDa at a heating time of 24 h. In addition, Shi et al. discovered that the lentil protein was hydrolyzed over the heating time from 0 to 30 h, and most of the protein hydrolysis occurred at an incubation of 5–8 h, which was related to the lag phase of fibrillization [38]. In Figure 3B, RA at pH 2 was disrupted into polypeptides with an Mw of 10–15 kDa, illustrating the high sensitivity towards acidic environments. As expected, the small peptides with an Mw of <15 kDa were observed during heating (0–24 h). This result revealed that the typical subunit composition of RA was destroyed by acid hydrolysis, and acidic conditions exhibited a more pronounced effect on protein hydrolysis than neutral conditions during heating. In addition, Wei et al. also found that the hydrolysis rate was accelerated when the pH shifted from 4 to 2 [17]. A possible explanation was that the abundant acidic amino acid residues (Glu and Asp) were more prone to degrade during acid heating [39]. As reported, amyloid core sequence and amino acid composition are of great importance in fibril formation [9]. For example, the vicilin fraction of legume protein was more prone to acid hydrolysis than legumin bands [16]. By comparison, the bands of RA were more inclined to degrade in acidic conditions. Hydrolysis of RA for 12 h at pH 7 and 90 °C yielded curly fibrils, while the spherical agglomerates were formed when the pH was adjusted to pH 2. This result revealed that the typical subunits of RA were destroyed by acid hydrolysis, and the excessive acid hydrolysis destroyed the fibril-core sequences of RA, which was not conducive to the ordered fibril formation. However, after heating at mild pH 7, the RA underwent a partial denaturation and assembly phase, of which internal bonds of proteins were broken, and active building blocks were produced and subsequently transformed to fibrils. Moreover, the superior hydrophilicity of RA at neutral conditions promoted the fibril formation [18].

### 3.4. CD Spectra

The fibrillization is commonly accompanied by structural changes. Since the formation of cross-β sheet structures is important for the fibril assembly process, the secondary structure alteration of RA during the heating process was monitored by CD spectra [4]. In Figure 3C, the CD spectra in the far-UV region (190–260 nm) of RA at pH 7 showed a profile with a negative dip at a minimum of ~206 and ~218 nm, suggesting that RA contained mainly random coil and β-sheet structures. Consistently, the random coil and β-sheet structures are 39.8% and 33.0%, respectively (Table S4), which were calculated by the BeStSel prediction algorithm. Similarly, it was found that the albumin from rice bran was rich in α-helix (26.44%), random coil (24.19%) and β-sheet structures (29.18%) [40]. After incubation, the negative peak of the samples showed a shift toward a lower wavelength, from 206 to 203 nm, indicating protein unfolding and fragment formation. And the secondary structure proportions of RA during the heating process are summarized in Table S4. During heating at pH 7, there was a decrease in β-sheet structure (from 33.0% to 24.6%) and an increase in random coil (from 39.8% to 49.6%) at a heating time of 0.5 h, indicating the unfolded structure of the protein, which was in line with the formation of short protofibrils with lower height. In addition, the β-sheet structure raised with prolonged heating time (1–24 h), indicating the reorganization of the protein structure and increased yield of fibrils, which was in agreement with the continuously increased ThT intensity. Consistently, the shorter protofibrils were elongated to longer flexible fibrils during fibrillization (Figure 2). This result was in line with our previous studies, which firstly observed a decreasing and subsequently increasing trend of β-sheet structure in rice glutelin during fibrillization [7]. In addition, other research also observed the increased antiparallel β-sheet structure of the protein after incubation [11,41], implying that the formation of the β-sheet structure was crucial for the fibrils self-assembly. With regard to pH 2, the CD profile of RA during the heating process is shown in Figure 3D. It can be seen that the ellipticity at 218 nm in the CD spectrum was significantly reduced during heating. Moreover, the negative peak at 207 nm was shifted to 200 nm, proving the disrupted structure of the protein and disordered conformation formation. As shown in Table S4, the α-helix of the protein declined obviously (from 25.30% to 5.4%); meanwhile, the β-turn and random coil of the protein were increased distinctly. However, no obvious alteration was observed in the β-sheet structure. This result demonstrated that the excessive acid hydrolysis of RA at pH 2 promoted the disordered structure formation, but inhibited the β-sheet structure re-arrangement. Presumably, heating at a mild pH resulted in the opening of the secondary structure, allowing the transformation of other structures to a β-sheet structure, thereby promoting the fibrillar aggregate assembly [18].

### 3.5. Average Particle Size

Particle size distribution is commonly regarded as an indicator of protein aggregation behavior [42]. The average particle size of RA at different pH values during the heating process is shown in Figure 3E,F. The average particle size of unheated RA at pH 7 (199.33 nm) was lower than that at pH 2 (279.25 nm), indicating that the proteins were prone to form larger particles in the acidic environment, which was in line with the AFM result (Figure 2). This was related to the lower solubility of RA at pH 2 (Table S1). In Figure 3E, an overall upward trend (ranging from 199.33 nm to 289.30 nm) was observed in RA at pH 7, illustrating that the interactions between proteins were enhanced and the aggregation of protein monomers occurred during fibrillization [25]. However, there was a reduction in size in the intermediate process (1–10 h), which was due to the denaturation and unfolding of the protein [43]. As shown in Figure 3F, the average particle size of RA at pH 2 slightly increased during the initial heating time (0–1 h), and then sharply increased to 3242.50 nm with the extension of heating time (2–6 h), attributing to the formation of spherical agglomerate (Figure 2). However, as the heating time increased (8–24 h), the average particle size decreased progressively, possibly due to excessive hydrolysis and degradation of the proteins. Overall, the average particle size of RA at pH 2 was higher than that at pH 7 during fibrillization. A previous study suggested that the particle size at 1000–6000 nm represented the large aggregation formation [44]. Thus, it can be concluded that RA was inclined to form larger amorphous aggregates during heating in an acidic environment. Considering the significantly reduced surface hydrophobicity during heating at pH 2 (Table 1), the formation of larger amorphous aggregates can be attributed to the hydrophobic interaction between protein particles [25]. Compared to the neutral condition (pH 7), the acidic condition (pH 2) approached the isoelectric point (pH 4.1), leading to reduced electrostatic repulsion and extensive aggregation [34]. Nevertheless, RA was capable of assembling ordered fibrillar aggregates during heating in the neutral solution.

### 3.6. Surface Hydrophobicity (H_0_)

The H_0_ is widely used to evaluate the hydrophobic groups on the surface of proteins [45]. The H_0_ of RA during heating at different pH values was measured using ANS as a fluorescence probe, which is shown in Table 1. The H_0_ of RA at pH 2 was higher than that at pH 7, indicating that there are more hydrophobic residues on the surface of the protein when the pH shifted from a neutral solution to an acidic environment. In addition, the greater surface hydrophobicity of the RA samples at pH 2 than at pH 7 was attributed to the acidic treatment dissociating the protein into smaller peptides, thereby exposing the hydrophobic groups and non-polar side chains [46]. After heating, the H_0_ of RA at pH 7 decreased obviously from 187.70 to 145.12, revealing that the hydrophobic patches were incorporated into the assembly of fibrils. Another explanation was that the formation of fibrillar aggregates involved hydrophobic interactions. Wan et al. also found that the H_0_ of soy protein declined after heating, indicating that the fibrillization was driven by hydrophobic interactions [34]. A similar downward tendency also occurred in RA at pH 2 during the heating process. The H_0_ of RA decreased by 81.78% at pH 2, which was higher than that of RA at pH 7 (29.34%), indicating that the protein exposed to an acidic solution strengthened the hydrophobic interactions between protein [47]. As reported, the balance between electrostatic repulsion interaction and attractive hydrophobic interactions played an important role in forming fibrils [7,48]. The pH value bears a twofold effect in fibril formation: cleavage of peptide bonds and modulation of electrostatic interactions [1]. Compared to the neutral condition (pH 7), the acidic condition (pH 2), approaching the isoelectric point (pH 4.1), led to reduced electrostatic repulsion and extensive aggregation [42], inhibiting the fibril formation. Therefore, it was concluded that the excessive hydrophobic forces and the decline of electrostatic repulsion in acidic solution between proteins broke the balance of the assembly process, inducing excessive aggregation, thereby inhibiting the ordered β-sheet structure and fibril formation.

## 4. Conclusions

RA is a promising substrate for manufacturing amyloid fibrils due to its sustainability. The formation of heating-induced amyloid fibrils derived from RA under neutral conditions was observed in this study. It can be seen that the ThT intensity of RA at pH 7 was enhanced throughout the process of fibrillization, and the optimal concentration of RA for forming amyloid fibrils was achieved at a concentration of 2%. In addition, worm-like fibrils with a contour length of 100–300 nm were formed after fibrillization, proving that the neutral condition was suitable for RAF formation. Given that the short and worm-like fibrils possess excellent interface stability, they can be applied as stabilizers of foams and emulsions. From structural characteristics, it was found that the RA underwent partial degradation of the polypeptides, the formation of a stable β-sheet structure and an increased particle size during fibrillization. Moreover, the process of fibrillization involved the balance of electrostatic repulsion forces, hydrophobic interactions and the conversion of a disordered state to an ordered β-sheet structure. Conversely, no fibrils were detected when RA was heated at pH 2, due to the fact that the excessive hydrolysis destroyed the fibril-core sequences of RA. Another explanation was that the low solubility of RA at pH 2 was not conducive to ordered aggregation. In summary, this work explores the suitable conditions for RAFs formation and analyzes the structural variation at different pH values, which will provide an insight into RAFs utilization in various environmental conditions. Future studies could investigate other factors, such as pre-fibril seeds and denaturants, to potentially accelerate the formation and regulation of the fine structure of amyloid fibrils and explore the application in food products.

## Figures and Tables

**Table 1:** The surface hydrophobicity of RA at different pH values.

Heating Time/h	Surface Hydrophobicity	
	pH 7	pH 2
0	187.70 ± 1.99 ^e^	606.19 ± 10.09 ^f^
0.5	182.23 ±1.16 ^d^	613.57 ± 2.79 ^f^
1	186.46 ±2.64 ^e^	612.48 ± 8.66 ^f^
2	180.29 ±2.03 ^cd^	542.00 ± 7.17 ^e^
4	178.07 ±2.09 ^c^	542.39 ± 8.34 ^e^
6	191.29 ±1.62 ^f^	508.51 ± 7.64 ^d^
8	182.13 ±1.81 ^d^	500.15 ± 8.14 ^d^
10	183.13 ±2.12 ^d^	436.27 ± 7.71 ^c^
12	164.12 ±0.38 ^b^	385.07 ± 6.29 ^b^
24	145.12 ±1.41 ^a^	333.48 ± 5.11 ^a^

Note: Data are exhibited as mean ± standard error. The different letters (a–f) mean a significant difference in the data (*p* < 0.05).

## Data Availability

The original contributions presented in this study are included in the article/Supplementary Material. Further inquiries can be directed to the corresponding author.

## Supplementary Materials

The following supporting information can be downloaded at: https://www.mdpi.com/article/10.3390/foods14173069/s1, Table S1: The solubility of RA at different pH values; Table S2: The maximum ThT intensity value and growth rate of RA at pH 7; Table S3: The length and height distribution of RA at pH 7 during heating; Table S4: The secondary structure content of RA at different pH values during fibrillization; Figure S1: The aggregation kinetics of RA with different protein concentration after heating at pH7; Figure S2: The AFM images of RA after heating at pH 7 for 6 h; Figure S3: The AFM images of RA after heating at pH 2 for 6 h.

## Abbreviations

The following abbreviations are used in this manuscript:

RA	rice albumin
ThT	thioflavin T
Glu	glutamic acid
Asp	aspartic acid
RAFs	RA-based fibrils
Mw	molecular weight
BCA	Coomassie Blue Staining Solution and bicinchoninic acid
ANS	8-Anilino-1-naphthalenesulfonic acid
AFM	Atomic force microscopy
SDS-PAGE	Sodium dodecyl sulfate polyacrylamide gel electrophoresis
CD	Far-UV Circular dichroism
H_0_	Surface hydrophobicity
SPSS	Statistical Product and Service Solutions
ANOVA	one-way analyses of variance
