# Supplementary material for: Formation and Structural Characteristics of Heating-Induced Amyloid Fibrils Derived from Rice Albumin at Different pH Values
Source: Foods. 2025 Aug 30;14(17):3069. doi: 10.3390/foods14173069 (PMC12428537; doi:10.3390/foods14173069)
Supplement: Supplementary file 1 [file foods-14-03069-s001.zip › foods-3827605-supplementary.pdf]

Supplementary materials

Table S1. The solubility of RA at different pH values.

Samples	Solubility (%)
pH 7	83.85±2.00
pH 2	32.53±1.13

Table S2. The maximum ThT intensity value and growth rate of RA at pH 7.

Samples at pH 7	Maximum ThT intensity (a.u.)	Growth rate (h^{-1})
1%	235.75±3.48	0.94
2%	532.19±4.55	1.37
4%	733.32±4.95	1.09

Table S3. The length and height distribution of RA at pH 7 during heating.

Heating time	Average length (nm)	Average height (nm)
0.5 h	78.83±10.62	4.63±0.91
6 h	112.17±29.56	4.68±1.20
12 h	199.74±86.89	5.09±1.04

Table S4. The secondary structure content of RA at different pH values during fibrillization.

	Heating time/h	α-helix	β-sheet	β-turn	random coils
pH 7	0	25.7	33.0	1.5	39.8
	0.5	21.5	24.6	4.3	49.6
	1	22.2	31.1	2.7	44.0
	2	21.1	26.7	3.9	48.3
	4	19.2	25.2	4.8	50.8
	6	19.5	27.5	4.3	48.7
	8	17.3	26.8	5.1	50.8
	10	17.2	27.4	5.0	50.4
	12	16.3	25.8	6.3	51.6
	24	15.9	29.6	5.5	49.1
pH 2	0	25.3	25.2	5.6	44.0
	0.5	22.3	27	6.2	44.5
	1	18.5	19.7	12	49.8
	2	18.7	26.6	7.9	46.8
	4	15.5	22.1	12.3	50
	6	15	22.9	12.4	49.7
	8	10.8	24.3	14.5	50.5

	10	7.7	25.3	14.7	52.3
	12	5.7	24.7	15.2	54.4
	24	5.4	25.9	16.3	52.4

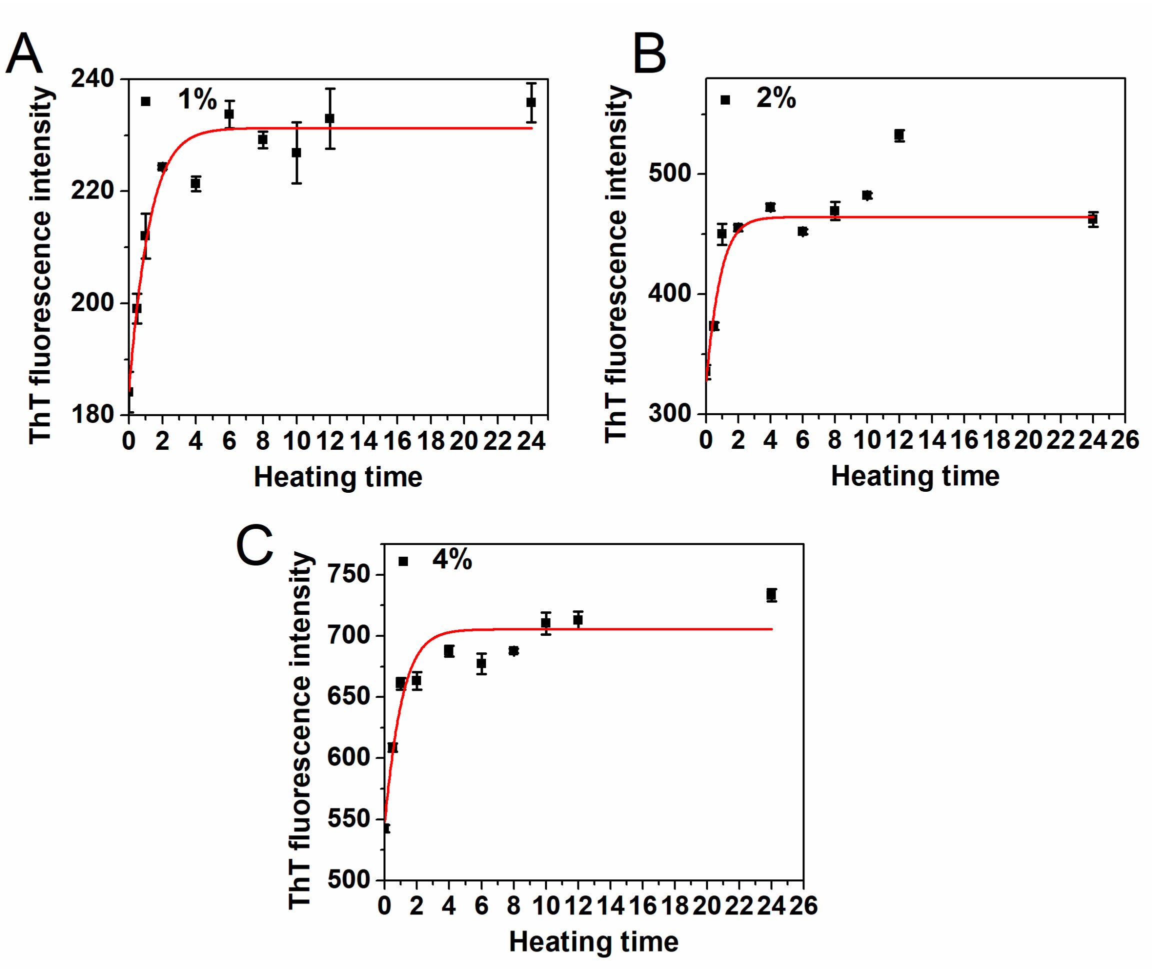

Figure S1. The aggregation kinetics of RA with different protein concentration after heating at pH 7.

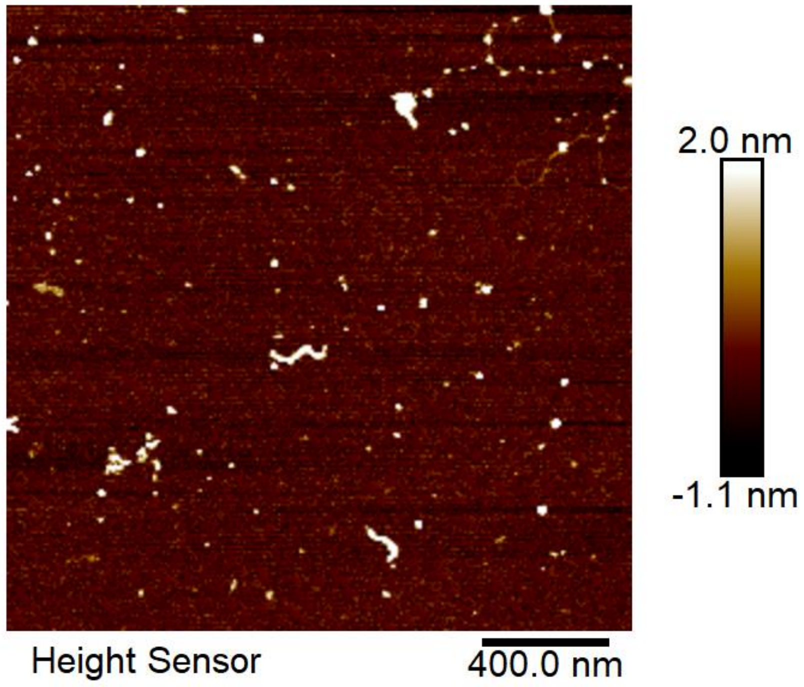

Figure S2. The AFM images of RA after heating at pH7 for 6 h.

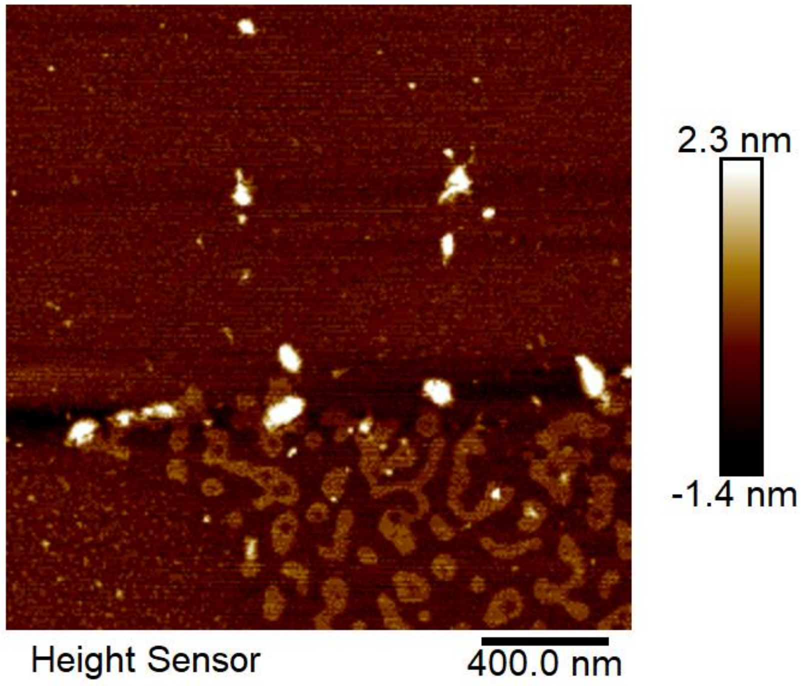

Figure S3. The AFM images of RA after heating at pH 2 for 6 h.
